# Assessment of the Perceived Acceptability of an Early Enrollment Strategy Using Advance Consent in Health Care–Associated Pneumonia

**DOI:** 10.1001/jamanetworkopen.2018.5816

**Published:** 2018-12-14

**Authors:** Amy Corneli, Brian Perry, Deborah Collyar, John H. Powers, John J. Farley, Sara B. Calvert, Jonas Santiago, Helen K. Donnelly, Teresa Swezey, Carrie B. Dombeck, Carisa De Anda, Vance G. Fowler, Thomas L. Holland

**Affiliations:** 1 Clinical Trials Transformation Initiative, Duke University School of Medicine, Durham, North Carolina; 2 Department of Population Health Sciences, Duke University School of Medicine, Durham, North Carolina; 3 Duke Clinical Research Institute, Duke University School of Medicine, Durham, North Carolina; 4 Patient Advocates in Research, Danville, California; 5 Department of Medicine, George Washington University School of Medicine and Health Sciences, Washington, DC; 6 Center for Drug Evaluation and Research, US Food and Drug Administration, Silver Spring, Maryland; 7 Department of Medicine, Northwestern University Feinberg School of Medicine, Chicago, Illinois; 8 Merck & Co, Inc, Kenilworth, New Jersey; 9 Department of Medicine, Duke University School of Medicine, Durham, North Carolina

## Abstract

**Importance:**

Better treatment options are needed in life-threatening infections, including health care–associated pneumonia. Enrolling patients in antibacterial clinical trials before diagnosis may circumvent existing time-to-enrollment constraints. However, the acceptability of an early enrollment strategy using advance consent is unknown.

**Objective:**

To assess the perceived acceptability of an early enrollment strategy for enrolling patients in an antibacterial clinical trial before a pneumonia diagnosis.

**Design, Setting, and Participants:**

This qualitative, descriptive study used semistructured telephone interviews. Framed within a planned noninferiority pneumonia antibiotic trial, an early enrollment strategy was described and perceptions were assessed. Using this strategy, patients give consent to enroll before developing pneumonia, to be monitored by study staff, and to be randomly assigned a study antibiotic if pneumonia develops. All interviews were audiorecorded, transcribed verbatim, and analyzed using applied thematic analysis. Fifty-two key stakeholders from across the United States, including 18 patients at risk of pneumonia, 12 caregivers, 10 representatives of institutional review boards, 7 investigators, and 5 study coordinators, were interviewed from June 20 to August 19, 2016.

**Main Outcomes and Measures:**

Perceived acceptability of the early enrollment strategy.

**Results:**

Among the 52 stakeholders interviewed (ages 29-75 years; 14 women), patients and caregivers expressed no concerns about patients being approached about participation before developing pneumonia; however, some patients may experience anxiety on learning about their risk for pneumonia. No concerns with study staff accessing patients’ medical records were expressed. The clarity of consent information was important for understanding the study rather than having the condition under investigation. Among patients, caregivers, and institutional review board representatives, preferences varied regarding opt-out and precedent autonomy procedures. Nearly all patients would be willing to join a trial using the early enrollment strategy and caregivers would be willing to provide proxy consent. Institutional review board representatives were supportive of the strategy and made recommendations for the study protocol, primarily around informed consent. Investigators and study coordinators believed the strategy would not be burdensome and offered suggestions to ensure its feasibility.

**Conclusion and Relevance:**

Results of the study suggest that the early enrollment strategy is acceptable. Future research should evaluate whether the strategy improves enrollment rates in registrational pneumonia trials and in trials of other acute infection syndromes with narrow enrollment windows and/or patients with transient decisional incapacity.

## Key Points

**Question:**

Is an early enrollment strategy using advance consent for research on health care–associated pneumonia acceptable to stakeholders?

**Findings:**

In this qualitative study of 52 stakeholders (patients at risk for pneumonia, caregivers, study investigators and coordinators, and representatives of institutional review boards), patients and caregivers found approaching patients and monitoring their records before they acquire pneumonia to be acceptable, indicated that patients can understand consent information before diagnosis, and described preferences for opt-out and precedent autonomy procedures. Institutional review board representatives were supportive of the strategy, and investigators and study coordinators indicated it would not be burdensome.

**Meaning:**

Results of the study suggest that an early enrollment strategy is acceptable to stakeholders and should be evaluated for effectiveness in increasing enrollment in registrational clinical trials.

## Teaser

This qualitative study assesses the perceived acceptability of stakeholders (patients, caregivers, investigators) to an early enrollment strategy using advance consent to antibacterial clinical trials for treatment of hospital-associated and ventilator-acquired bacterial pneumonia.

## Introduction

On any given day, approximately 1 in 25 patients in US acute care hospitals have a health care–associated infection. Two of the most common infections are hospital-acquired and ventilator-associated bacterial pneumonia (HABP/VABP).^1^ Pneumonia caused by susceptible and resistant pathogens is associated with the most infection-related deaths in the United States and is the eighth leading cause of death overall.^2^ However, high-quality clinical trials to identify antibiotics for HABP/VABP are difficult to conduct and prohibitively expensive, costing upwards of $90 000 per enrolled patient, primarily because of the large number of patients who must be screened for eligibility.^3^ This apparent paradox between common infections and infrequent trial eligibility is in part attributable to difficulty in identifying patients, confirming HABP/VABP, and obtaining informed consent from patients who urgently need treatment and from very ill patients who may no longer be able to consent for themselves.^4,5^ Moreover, prior effective antibacterial drug therapy may limit the ability to assess the effect of a new antibacterial drug in a noninferiority clinical trial.^6,7,8^ These factors lead to a very limited window for enrollment.

As part of its project portfolio on antibacterial drug development,^9^ the Clinical Trials Transformation Initiative, a public-private partnership cofounded by the US Food and Drug Administration (FDA) and Duke University, Durham, North Carolina, held multistakeholder meetings to address challenges in conducting HABP/VABP clinical trials and to explore alternative enrollment models.^8,10^ A novel early enrollment strategy emerged from these discussions, whereby patients at high risk for HABP/VABP while in the hospital are asked to provide advance consent to participate in clinical research—that is, they are enrolled in a clinical research study on HABP/VABP before being diagnosed with either type of pneumonia. Patients consent to monitoring by study staff and, if HABP/VABP develops, to being randomly assigned to receive an investigational or a comparator antibiotic. Advance consent is typically used in situations when individuals (1) may lose their decision-making capacity^11^ or (2) are at high risk for a condition for which therapy must be initiated without delay.^12^ Clinical trials for HABP/VABP fall within the latter condition, in which research is limited; almost all research on advance consent focuses on persons with dementia or on broad advance research directives.^11,13,14,15,16,17,18,19^

The Clinical Trials Transformation Initiative planned to conduct a pilot study to evaluate the effectiveness of early enrollment using advance consent in a noninferiority antibiotic treatment trial. However, because questions about acceptability remained, we conducted formative research to assess the acceptability of the early enrollment strategy among key stakeholders.

## Methods

### Design

We conducted a qualitative, descriptive study using semistructured telephone interviews with the following 5 key stakeholder groups involved in HABP/VABP clinical research: patients, caregivers, representatives of institutional review boards (IRBs), investigators, and study coordinators. Interviews were conducted from June 20 to August 19, 2016. The IRB of the Duke University Health System determined that the research met the requirements for exemption from further IRB review. Written informed consent was not required. Instead, all participants received an information sheet before study participation that explained the study in detail, including its purpose, risks, and benefits.

### Participant Selection, Eligibility, and Recruitment

We used purposeful sampling^20^ to select participants who were at risk of HABP/VABP (ie, patients), had a family member at risk of HABP/VABP (ie, caregivers), or had experience with research on HABP/VABP (ie, IRB representatives, investigators, and study coordinators). The eMethods in the Supplement provides more information about eligibility and recruitment.

### Data Collection

We began each interview by describing the early enrollment strategy. We then described the antibiotics to be used in the treatment phase of the research. After describing all study-related information and answering participants’ questions, we asked questions about the acceptability of the early enrollment strategy. Constructs of acceptability were informed by an established feasibility framework^21^ and varied by participant group (Table 1). We used a vignette-based approach^22^ to explore participants’ perceptions of opt-out and precedent autonomy^23^ procedures. All interviews were audiorecorded with the participants’ permission. The eMethods in the Supplement provides more information about data collection.

**Table 1.** Acceptability Measurements by Participant Group

Participant Group	Construct	Measurement
Patients and caregivers	Appropriateness	Reactions to being approached to consider trial participation for a condition not yet diagnosed
		Reactions to being informed of risk of a life-threatening condition
		Beliefs about whether patients can understand the research without having the condition under investigation^12^
		Perceptions of opt-out procedures, including whether patients’ initial consent should remain valid if they subsequently lose their ability to opt out at the time of randomization (ie, precedent autonomy)
	Intent	Perceptions of willingness to (1) enroll in a trial in advance of developing the infection under investigation, within the context of a noninferiority clinical trial, and (2) allow study staff to monitor health records
Investigators and study coordinators	Feasibility	Perceptions of level of burden from using an early enrollment strategy with advance consent
IRB representatives	Appropriateness	Perceptions of (1) any ethical or regulatory concerns about an early enrollment strategy with advance consent, and (2) opt-out procedures

Abbreviation: IRB, institutional review board.

### Data Analysis

We use descriptive statistics to summarize characteristics of the study participants, and we used applied thematic analysis^24^ to analyze participants’ narratives. All interviews were transcribed verbatim following a standardized transcription protocol.^25^ We used NVivo qualitative data analysis software (version 11; QSR International Pty Ltd) to apply structural codes to segment participants’ narratives into conceptual categories based on the topics explored in the interviews. We assessed intercoder reliability on at least 1 interview from each participant group; we discussed discrepancies, revised the codebook, and recoded transcripts when necessary. Next, we inductively identified and applied content-derived codes to the narratives in each structural coding report, allowing for identification of issues salient to participants. We then organized content codes thematically, depending on relationships between codes. Summary reports described the major themes and subthemes and provided illustrative quotations.

## Results

We interviewed 52 key stakeholders from across the United States, including 18 patients at risk of HABP/VABP, 12 caregivers of patients at risk of HABP/VABP, 10 IRB representatives, 7 investigators, and 5 study coordinators. Among patients, a range of ages were represented (29-75 years), 14 were women, all 18 self-identified as white, and 10 had a bachelor’s degree or higher. Among caregivers, 9 ranged in age from 45 to 64 years, 8 were women, 10 were white, and all 12 had at least a high school education (Table 2).

**Table 2.** Characteristics of the Patient and Caregiver Participants

Characteristic	Participant Group, No. (%)	
	Patients (n = 18)	Caregivers (n = 12)
Age group, y		
25-44	5 (27.8)	2 (16.7)
45-64	5 (27.8)	9 (75.0)
≥65	8 (44.4)	1 (8.3)
Sex		
Male	4 (22.2)	4 (33.3)
Female	14 (77.8)	8 (66.7)
Race/ethnicity		
Black or African American	0	2 (16.7)
White	18 (100)	10 (83.3)
Non-Hispanic/non-Latino	18 (100)	12 (100)
Educational attainment		
Some high school (grades 9-12)	1 (5.6)	0
High school diploma or equivalent	2 (11.1)	2 (16.7)
Some college credit	3 (16.7)	3 (25.0)
Associate’s degree	2 (11.1)	3 (25.0)
Bachelor’s degree	3 (16.7)	0
Master’s degree	7 (38.9)	3 (25.0)
Doctorate or professional degree	0	1 (8.3)
Health status		
Been in the ICU or had overnight hospital stay and previous HABP/VABP diagnosis or at-risk condition^a^	17 (94.4)	12 (100)
Has chronic lung disease	16 (88.9)	6 (50.0)

Abbreviations: HABP/VABP, hospital-acquired and/or ventilator-associated bacterial pneumonia; ICU, intensive care unit.

^a^ Patients responded with reference to their own health status; caregivers responded with reference to the patient’s health status.

Investigators were all from different institutions, as were the study coordinators and IRB representatives (except for 2 IRB members—1 community member and 1 scientific member—who were from the same university). All investigators were participating as site investigators in the associated Predicting Pneumonia in Hospitalized Patients in the ICU (PROPHETIC) study—a prospective observational cohort study conducted at 28 intensive care units (ICUs) in the United States to determine the proportion of at-risk patients who develop pneumonia^26^—and had experience in conducting phases 1 through 3 clinical trials. Three study coordinators were also involved in the PROPHETIC study, and 2 served as coordinators in other ICU-based studies. Among the IRB representatives, 6 were IRB chairpersons, 2 were scientific members, and 2 were community members; 8 had 5 or more years of experience as IRB representatives.

### Appropriateness

#### Patients and Caregivers on Being Approached About Trial Participation Before Diagnosis

Patients and caregivers expressed no concerns about being approached in the ICU about a clinical trial on treatment for pneumonia before the patient was diagnosed with the condition. Several explained that they were not concerned because (1) they already knew their own or the patient’s risk for pneumonia in general or when people are hospitalized; or (2) they understood that, given their own or the patient’s underlying health conditions, being asked to consider a trial on pneumonia specifically, in comparison with other unrelated conditions, would not seem unrealistic (concept 1, Table 3).

**Table 3.** Patient and Caregiver Perceptions of Learning About Being at Risk of Pneumonia, of Patients Joining a Study Before Diagnosis, and of Trial Comprehension

Concept		Excerpt
1. Concerns about joining a study before diagnosis		“No, I don’t think so [be concerned]. I think because of the situation you’re in. You’re in the ICU. It’s been proven that this [pneumonia] can develop…I don’t think that that’s concerning at all. I wouldn’t take concern with it.”—Female patient, 20s
		“No [have concerns], but see, this is the point…They [patient] have COPD and because of that, there is a risk of infections, and I know that.”—Male caregiver, 60s
2. Learning about being at risk for pneumonia: patients’ responses		
Have anxiety		“That would scare me to begin with…I mean that it’s always scary to go into a hospital to get better and know that you may get worse because of something that you may catch in the hospital.”—Female patient, 40s
Not surprised		“Well, me, personally, I would totally understand it because I have a lung condition.”—Female patient, 60s
Glad to be informed		“I would appreciate it. I like to have all the information that’s going on with my health.”—Female patient, 40s
Want to know plan to reduce risk		“It might be initially a little bit stressful, but then I’d also feel like I was informed and that…my health team and me were going to be making sure that I didn’t get pneumonia, but if I did, there’s a plan in place to get these certain drugs…”—Female patient, 30s
3. Learning about pneumonia risk: caregivers’ responses	“I would feel concerned, of course, but I wouldn’t really be surprised...it just puts more fear in you, but on the other hand, you want to know about the risks…you’re in a very vulnerable place, but…you appreciate being told what’s up…and I expect that, as part of it…whoever’s telling you the person…they’re saying, ‘These are the things that we do to prevent that from happening.’”—Female caregiver, 60s	
	“Personally, I would appreciate that. Yes, it would worry me. However, it would be nice to know those things and not get blindsided when it happened that this possibility is there…and this is what we would like to do prior to it happening, so you are aware of it.”—Female caregiver, 50s	
4. Trial comprehension: patients’ and caregivers’ responses		
Ability to understand the study		“I don’t know that that matters…whether you have pneumonia or you don’t have [it], at the time that you’re making your decision…if you suddenly have pneumonia, the information is the same.”—Female patient, 70s
		“Well, I understand why you’d be asking, even if I don’t have [pneumonia].”—Female patient, 70s
Information disclosure about study, in general		“I’m okay [with being asked to join a study for an infection I do not have]. I just need to know more about the study, which means I’d like to have the study coordinator come in and give me a thorough explanation of what the intent of the study is and what information they’re looking for…And if I’m ok with that, I’m glad to be part of the trial and help out with the goals of the study.”—Male patient, 70s
		“If I was given enough information about it, I should be able to [understand the study without actually having pneumonia].”—Female patient, 30s
Information to provide about risk		“I think that there needed to be a little more explanation…that pneumonia is a very common side effect for being in the hospital even if you don’t or never have had pneumonia. It can be, you being elderly or whatever, are at high risk for developing pneumonia.”—Female patient, 60s
Broader health context		“I think that could be a barrier [not having pneumonia], because they [patients] don’t have it and so it’s not something that’s on the forefront of their mind. They’re obviously in there [ICU] for another reason, and they’re probably really thinking, putting all of their energy into fighting whatever it is that’s going on right at the moment. So, that kind of creates a bit of a challenge, because if you’re in the mindset of prioritizing your health issues, you’re certainly not going to be thinking about what could happen. You’re thinking about what you’ve already been fighting, what you’ve already been going through. So, it may not be the priority for people to think about and talk about pneumonia if they don’t have it.” —Female patient, 40s
		“I don’t think that’s the difficulty [patient not having pneumonia at time of consent]…I think the difficulty is you’re in a very high emotional state because your loved one, family member, or the patients themselves are in acute distress, otherwise they would not be in an ICU.”—Female caregiver, 40s

Abbreviations: COPD, chronic obstructive pulmonary disease; ICU, intensive care unit.

#### Patients’ and Caregivers’ Reactions to Being Informed of Being at High Risk for Pneumonia

Approximately one-third of patients believed they or other patients would experience anxiety after learning about their risk of pneumonia. However, just as many patients said learning about their risk would not be surprising given their current health condition, and even more said they would appreciate being informed about the risk. Several patients added that it would be important to inform patients about the health care team’s plan to reduce the patient’s risk (concept 2, Table 3).

Although some acknowledged concern, caregivers expressed less anxiety about learning of a patient’s risk of pneumonia than the patients did. Caregivers’ narratives primarily focused on their current awareness or understanding of the risk and the importance of being informed of and having a plan to reduce the risk (concept 3, Table 3).

#### Patients and Caregivers on Trial Comprehension

Nearly all patients and caregivers said that not having pneumonia at the time of advance consent would not influence their or another patient’s ability to understand consent information. Patients’ narratives primarily focused on appropriate disclosure of information about the trial as the key to patients’ understanding, rather than the embodiment of the specific condition under investigation. However, several patients emphasized the importance of the risk context. For those who are aware of their pneumonia risk, understanding the study would not be difficult; for those who are unaware, they must first be informed of their risk to facilitate understanding.

Some patients and caregivers indicated that the patient’s broader health context—that is, their current medical condition and generally being very ill—could pose barriers to understanding or the patient’s or caregiver’s ability to focus on the consent information. They explained that the priority of patients in the ICU will be on their current critical illness, and not on other conditions they may acquire during hospitalization. In addition, a few patients believed that some patients may need more time to consider their participation if just learning about their pneumonia risk (concept 4, Table 3).

#### Patients, Caregivers, and IRB Representatives on Opting Out and Precedent Autonomy

Participants’ responses are situated within the context of designing a specific opt-out procedure when advance consent is used. Participants’ views varied on whether study staff should actively confirm trial participation wishes from patients who develop pneumonia before giving the study antibiotic or proceed with randomization on diagnosis and provide the study antibiotic without patient confirmation, with the caveat that participants can opt out of research at any time. Almost twice as many patients said “a conversation” with the patient should happen to confirm that study participation is still preferred, compared with patients who said study staff should proceed with randomization without confirmation. Similarly, more IRB representatives said patients’ previous consent should be confirmed at the time of randomization than said that no confirmation was necessary. With this confirmation approach, participants said that study staff should inform the patient that they have developed pneumonia, remind them of their advance consent, explain again the next steps, and confirm their agreement to proceed with randomization. However, some of these IRB representatives said they chose this approach only because investigators were designing an opt-out component to include in the study design; otherwise, they believed study staff should proceed with randomization because of the patient’s right to withdraw from research at any time (concept 1, Table 4).

**Table 4.** Opting Out and Precedent Autonomy

Concept	Excerpt
1. Opting out: confirm participation before randomization	“I think it should be presented to [the patient] as, ‘You have been diagnosed with pneumonia. The doctors want to treat it. You have signed up for this study, which means you get drug A or B. Are you still okay with participating in the study to treat your pneumonia?’”—Female patient, 60s
	“I think a conversation should be had with the subject, reminding them that they agreed to participate in this study, a description of the study and the drugs involved, the status of their disease, and confirm that now that they are at the point of having disease, that they do want to participate, they do want to continue with the treatment regimen on study.”—IRB community member 2
	“Well, [automatic randomization] would be what would happen without any opt-out process, because there’s always an opt-out thing in human subject research…If you’re going specifically [to] say people have the option to opt out, and highlight that, I suppose you ought to bring it up and ask them.”—IRB chairperson 1
2. Opting out: proceed with randomization without confirmation	“I think [the patient] already understands that she’s enrolled, she’s going to get drug A or B. I think that part is clear, and doesn’t need any more explanation…We got her consent before she was diagnosed, now she is sick, and we’re going to bring in the study person and say, ‘Okay, you have pneumonia, we’re going to treat you with [a study antibiotic] as we discussed earlier’…with reassurance that we’ll be monitoring you very, very, very, closely. ‘And, if we don’t see the results we want to see we’ll be making some changes.’”—Female patient, 60s
	“I think at that point, they start treatment, they go on [to] basically treat, you don’t go back and say do you want to opt out again. You don’t want to introduce that. It’s only if the patient comes back or the caregiver says no, we want to opt out. They made the decision. They did it already once.”—Male caregiver, 60s
	“If somebody gets an infection, it may be appropriate to inform them that they’ve potentially moved on to the next part of the study…not necessarily saying, ‘Hey, you don’t have to participate anymore,’ but saying, ‘Hey, remember, you signed this form, and you’ve got this infection. So we are randomizing you.’ Just to inform them of what’s happening in terms of the study. But not necessarily saying, ‘Hey you’ve moved on. Do you still want to participate?’…It’s not necessarily having to ask their permission to continue with something they’ve already agreed to, that they’ve already given permission for.”—IRB chairperson 2
3. Precedent autonomy^a^	
Proceed with randomization	“She was of sound mind and body when she agreed to participate.”—Female patient, 60s
	“Immediately give her the medication because she already agreed to it, so she knows what it’s about…just go ahead and give it to her. She already consented for it.”—Female patient, 50s
	“Just because she’s unconscious, doesn’t mean that her original decision doesn’t stand. Now, [even] if her husband was adamant, he was not interested in the clinical trial, I’m still going to have to go back to, unfortunately, she signed off on that. And, if you’ve got to make a phone call to find out what the final decision is, you’re just putting off giving them much needed medicine. You’d want to encourage [the patient], when she was given the opportunity to enroll in the trial, to discuss that with [her caregiver].”— Male caregiver, 40s
	“But in this instance, they’ve already given consent, they’ve already told you what they’re willing to do, and it shouldn’t be, quite frankly, shouldn’t be up to the LAR to change that decision.”—IRB chairperson 2
Inform LAR without ability to change patient’s initial consent	“[The LAR] should be notified of what is going to be happening, but if [the patient] gave the okay beforehand, then [the physicians] should go with her approach; they should go ahead and do what was explained to her…it wouldn’t be up to the [LAR] then because she had previously decided to consent.”—Female patient, 40s
LAR should opt out the patient	“She had thought about it before and said okay. She’s trusted him to give him the authority; he can evaluate what state she’s in. And because she can’t decide now, he can choose to opt out or stay in, depending on what feels right to him.”—Female patient, 60s
	“I think since she is unconscious that [the LAR] should be contacted and talked to and let him know exactly what’s going on regarding her being unconscious and developing pneumonia so he knows to make that informed decision whether or not he wants to continue.”—Female caregiver, 50s
	“Their LAR, the people closest to them are ultimately going to be, in a sense, responsible for making decisions from then on…I would think they would have a copy of the consent that was used for that particular study, and that they would go over with the LAR, and let them know, ‘Your loved one signed this on X date, and are you willing to allow this to go on?’”—IRB scientific member 1
It depends on whether study has specific opt-out procedures	“I think the answer to that depends on knowing exactly what the words are in the consent form of that opt out, because if the wording is such that you will be presented with the option to opt out before randomization, then the correct answer is to contact the LAR. Because, if somebody is unconscious, then the LAR acts on their behalf. On the other hand, if the opt-out language is simply saying that you have the option to opt out of research at any time, including before randomization… and it’s just a reminder of that, but there’s no statement that you will be specifically asked about it before we randomize you, and you’ll have to say that you want to stay in the trial, then I would say just randomize. It really depends on the exact wording of the consent form, on that opt-out paragraph.”—IRB chairperson 1
Explain process in consent form	“I would suggest they don’t have an opt out. I think that you explain it upfront. And, people, once they’ve consented to the study and the study activity, the fact that they lose capacity doesn’t overrule their prior decision.”—IRB chairperson 5

Abbreviations: IRB, institutional review board; LAR, legally authorized representative.

^a^ Indicates in cases where patients have become incapacitated after advance consent.

For those who believed that confirming participation on diagnosis was unnecessary, most said study staff should inform patients that they have developed pneumonia and proceed with randomization, per the advance consent that patients provided. One IRB representative, a chairperson, expressed that confirming the agreement defeats the purpose of using an early enrollment strategy. Caregivers were almost equally divided between the 2 approaches (concept 2, Table 4).

Perspectives also varied on precedent autonomy, that is, how to proceed when patients initially give advance consent but are unconscious at the time of randomization. About the same numbers of patients and caregivers said randomization should proceed based on the patient’s initial consent, compared with those who said a legally authorized representative (LAR) should have the option to withdraw the patient. Nearly all patients and caregivers who supported the patient’s initial consent suggested that LARs should be informed at the time of randomization that patients will be treated with a study antibiotic, and many said LARs should not be allowed to change the patient’s initial consent. Several patients acknowledged possible conflicts in situations where LARs do not want randomization to continue and suggested LARs be informed of the patient’s wishes at the time of advance consent to avoid future complications. Among patients and caregivers who said LARs should have the option to opt out patients, most stressed framing the discussion around the patient’s initial wishes. None of the patients or caregivers said standard treatment should be given. A few patients and caregivers suggested the consent form should explain procedures that will be followed if the patient becomes incapacitated or should indicate patients’ opt-out preferences if they subsequently become unconscious.

Among IRB representatives, just more than half said patients who become incapacitated before randomization should continue to randomization if pneumonia develops, based on their initial consent. They stressed that these patients previously gave informed consent for randomization if they were to develop pneumonia, making confirmation from LARs unnecessary. In addition, several said LAR confirmation “makes logistics more complicated.” A few IRB members said LARs should have the option to opt out the patients, although some acknowledged that they made their choice because the trial design included a specific opt-out component and, therefore, the study must provide a specific process for opting out. Several also discussed that the consent form should document patients’ wishes should they become unconscious or state the study procedures for when patients become unconscious (concept 3, Table 4).

#### IRB Representatives on Ethical and Regulatory Concerns

None of the IRB representatives expressed concern with the early enrollment strategy from an ethical and regulatory perspective. Half stated the strategy was “pretty straightforward” and “very reasonable” (section 1, Box). Without prompting, nearly half also offered advantages to using the early enrollment strategy, such as providing patients with more time to consider participation and obtaining patients’ consent before they become too sick (section 2, Box).

Box. **Institutional Review Board (IRB) Representatives’ Perceptions of the Early Enrollment Strategy Using Advance Consent and Its Advantages**

**Section 1: Overall Perceptions**

“Well, this sounds like a simple study. I’m not seeing any real issues it raises.”—IRB chairperson 1
“It is reasonable for somebody to consent to something ahead of time, before they might have [pneumonia].”—IRB chairperson 5
“Honestly, this sounds fairly straightforward. It doesn’t sound like it’s going to cause a great deal of concern.”—IRB community member 2

**Section 2: Advantages**

“I actually think it’s somewhat kinder to ask somebody to enroll in this kind of trial before they get the infection so they have time to consider it…rather than at the time they’re acutely ill and may be needing ventilation…I really like it for that reason.”—IRB chairperson 3
“It almost seems easier to fathom that people would have the opportunity to really think through the consent and decide if they want to participate or not [compared with] the patient who’s already acutely ill, where they’re trying to make a decision at that moment, and then they may feel more pressure, as opposed to the situation where there is less time pressure to make the decision about whether to be in the study or not.”—IRB chairperson 4
“The advantage is you give individuals, presumably, a longer run-up time to actually think about whether they would want to be enrolled in it, and there is also a higher likelihood that the actual subject would be able to consent as opposed to a legally authorized representative…one would think they would be more likely well enough to be able to consent for themselves. So I think it has—it has a number of advantages from that standpoint. —IRB chairperson 2

Several IRB representatives said investigators must ensure that patients (1) are capable of providing informed consent when in the ICU, (2) fully understand they can withdraw at any time, and (3) are provided with an “appropriate and adequate” consent form, particularly because patients are informed that they are at risk of a life-threatening condition (which could cause anxiety) and asked to provide their consent for a condition they have not experienced. Several IRB representatives added that a process for engaging patients’ LARs should be developed, especially in situations when patients lose consciousness between giving advance consent and randomization.

### Feasibility

Nearly all investigators and study coordinators believed the early enrollment strategy would not be burdensome to implement, although several acknowledged that there would be a large number of patients to be consented and monitored who might not develop HABP/VABP, making it “an inefficiency, but not a burden.” Some highlighted critical factors necessary for the early enrollment strategy to be feasible, primarily using appropriate eligibility criteria, collecting limited data before randomization, and budgeting realistically for staff time.

### Intent

#### Patients and Caregivers on Willingness to Enroll

When envisioning themselves in the ICU, nearly all patients and caregivers said they would be willing or likely willing to enroll or to give their proxy consent, respectively, in an early enrollment noninferiority clinical trial that evaluated 2 commonly used antibiotics for treating HABP/VABP (an FDA-approved antibiotic for pneumonia and an FDA-approved antibiotic for other serious infections but not pneumonia). Of those who said they would not provide their consent, their reasons were related to the treatment phase of the trial and not to the early enrollment strategy or advance consent.

#### Patients’ and Caregivers’ Willingness to Allow Study Staff to Monitor Health Records

None of the patients and caregivers expressed concern about monitoring of patients’ hospital records by study staff (although staff typically does not have such access) to determine whether patients develop pneumonia while in the ICU. Several patients and caregivers explained that their lack of concern was because they perceived it as commonplace for multiple health-care personnel to access patient records, that study staff are professionals, that the monitoring of patient records was in the patient’s best interest, that reviewing patient records was necessary to conduct the study, and that the information learned would be kept confidential and used for study purposes only.

## Discussion

Our findings suggest that an early enrollment strategy with advance consent is acceptable among key stakeholders. Patients and caregivers reported that approaching patients for research participation before a diagnosis of HABP/VABP was appropriate, given the context, but informing them of their risk for the condition may be concerning to some. Information disclosure was described by patients and caregivers as critical to understanding consent information, rather than the fact of having the condition under investigation. Patients and caregivers believed they would be willing to allow study staff to review the patient’s medical records before diagnosis and to give their consent to participate in the proposed noninferiority trial that used an early enrollment strategy.

The IRB representatives expressed no ethical or regulatory concerns with the early enrollment strategy using advance consent and provided advice on study procedures to incorporate when the strategy is used. Investigators and study staff acknowledged the additional workload of obtaining consent from potential participants, but they believed an early enrollment strategy would be feasible to implement. One critical consideration in moving forward with an early enrollment strategy is the ratio of patients screened to those enrolled, that is, how many patients would need to be approached for early consent to yield 1 patient enrolled in an interventional study. The PROPHETIC study mentioned previously is part of an effort to define rates of incident pneumonia among specific subpopulations, which will enable a targeted approach.^26^

Variation in participants’ perceptions were observed regarding opt-out and precedent autonomy procedures. For future studies using the early enrollment strategy, findings from additional research with the study population or guidance from community advisory structures may indicate the best opt-out approaches to use. With precedent autonomy, documenting the patient’s wishes at the time of consent and sharing this information with the patient’s LAR may be appropriate if the patient is willing to disclose their study participation.

### Limitations and Strengths

Our findings should be considered within the context in which the data were gathered. Because we interviewed investigators, study coordinators, and representatives from IRBs who were engaged in the PROPHETIC study, they may have been more accepting of the proposed early enrollment strategy to HABP/VABP clinical trials than those without this experience. However, their responses were grounded in actual HABP/VABP research experiences, which we believe better inform perceptions of the acceptability of the early enrollment strategy compared with hypothetical responses from individuals without this experience. In addition, as is typical with formative research, we purposely selected patients who were similar to patients enrolled in HABP/VABP interventional studies and likely similar to those who will participate in the future research. Although we anticipate similar risk factors between the formative and future study populations, the demographic characteristics could differ. As with all formative research, a limitation is that participants describe their perceptions of how they believe they would respond to certain situations; once they actually experience the situation, their viewpoints may change. Nonetheless, formative research provides useful information in making decisions about moving forward with clinical research by ensuring that aspects are included that stakeholders believe will be important. In our case, we learned that the early enrollment strategy with advance consent is an acceptable approach.

Although our use of the early enrollment strategy focuses on HABP/VABP, this approach also may be applicable to trials in other conditions for which time is of the essence and/or recurrent episodes of decisional incapacity occur. For example, patients who are at risk of HABP/VABP are also at risk of other ICU-acquired infections, such as bloodstream infections, complicated urinary tract infections, and *Clostridium difficile* colitis. Similarly, trials with patients with noninfectious chronic conditions with frequent exacerbations—eg, sickle cell disease or asthma—could also use this strategy. Trials on conditions with recurrent, temporary decisional incapacity, such as patients with cirrhosis who are prone to recurrent bouts of hepatic encephalopathy, could also use an early enrollment strategy at a time when the patient is able to meaningfully participate in consent discussions.

## Conclusions

The early enrollment strategy with advance consent appears to be an acceptable approach among key stakeholders. Given its potential to improve trial feasibility and to generate urgently needed data to improve patient care, our findings suggest that sponsors should evaluate whether the early enrollment strategy improves enrollment rates in registrational HABP/VABP clinical trials. Qualitative research should be embedded into such trials to explore patients’ and caregivers’ reactions to the strategy in real-world settings.

## Notes

Supplement. **eMethods.** Information on Participant Eligibility, Recruitment, and Data Collection
