# Supplementary material for: Assessment of the Perceived Acceptability of an Early Enrollment Strategy Using Advance Consent in Health Care–Associated Pneumonia
Source: JAMA Netw Open. 2018 Dec 14;1(8):e185816. doi: 10.1001/jamanetworkopen.2018.5816 (PMC6324354; doi:10.1001/jamanetworkopen.2018.5816)
Supplement: Supplement. — eMethods. Information on Participant Eligibility, Recruitment, and Data Collection [file jamanetwopen-1-e185816-s001.pdf]

Supplementary Online Content

Corneli A, Perry B, Collyar D, et al. Assessment of the perceived acceptability of an early enrollment strategy using advance consent in health care–associated pneumonia. *JAMA Netw Open*. 2018;1(8):e185816. doi:10.1001/jamanetworkopen.2018.5816

eMethods. Information on Participant Eligibility, Recruitment, and Data Collection

This supplementary material has been provided by the authors to give readers additional information about their work.

© 2018 Corneli A et al. *JAMA Network Open*.

eMethods. Information on Participant Eligibility, Recruitment, and Data Collection

Information About Participant Eligibility and Recruitment

We purposely selected participants based on being at risk of HABP/VABP (patients), having a family member at risk of HABP/VABP (caregivers), and having had experience with research on HABP/VABP (investigators, coordinators, and IRB members). Patients were eligible if they had been in the intensive care unit (ICU) or stayed overnight in a hospital at least once in the past 2 years and had a previous diagnosis of HABP/VABP or an illness or event that would place them at risk for HABP/VABP (ie, acute illness, chronic illness, physical trauma, or surgery, or are immunocompromised). Patients were also eligible if they had chronic lung disease without previous hospitalization. Caregivers were eligible if they were an adult caregiver for an individual who met the patient eligibility criteria. Almost half of the patients were recruited through a professional recruitment firm. The remaining patients were recruited from a patient group Facebook page; 2 were recruited through provider and flier recruitment at a university. No patient who was currently participating in the PROPHETIC study was directly recruited to participate in the study. Nearly all caregivers were recruited by the professional recruitment firm; 1 caregiver contacted study staff after learning about the study from a patient participant in this study.

Investigators and study coordinators were eligible if they were a site investigator or study coordinator, respectively, on the associated PROPHETIC study. Study coordinators were also eligible if they had been a coordinator for an ICU-based study. Institutional review board (IRB) members were eligible if they were a chair or voting member (scientific or community) and their IRB was associated with the PROPHETIC study. Investigators, study coordinators, and IRB

© 2018 Corneli A et al. *JAMA Network Open.*

members received individual email invitations to participate from study team members. We identified investigators, study coordinators, and IRB representatives to invite for participation in the interviews so we could have representation from across the United States and from different institutions.

Information About Data Collection

Demographic Data Collection

Demographic information was collected before the interviews and is reported in Table 2 in the manuscript.

Interview Context

Patients and caregivers were mailed cue cards to view during the explanation of the early enrollment strategy and treatment trial to aid with understanding. They were also provided with a rationale for the early enrollment strategy. The summary for IRB representatives was designed to be similar to summaries given by primary reviewers during IRB meetings. The interviewers did not personally know any of the study participants.

Explanation of Study Antibiotics

We explained that 1 antibiotic (imipenem) to be used in the trial has been FDA-approved for lower respiratory infections (ie, pneumonia) and the other antibiotic (meropenem) had been FDA-approved for other serious infections, but not for pneumonia, although it is commonly used off-label in hospitals throughout the United States as a treatment for pneumonia. We also

© 2018 Corneli A et al. *JAMA Network Open.*

explained that meropenem might not work in treating pneumonia as effectively as imipenem but might have fewer side effects.

Vignettes

In the interviews with patients and caregivers, we used a vignette-based approach to frame questions on opting out and precedent autonomy. In this vignette, Susan—a 57 year old married woman—has been admitted to the ICU at a local hospital. She does not currently have pneumonia but is considered at high risk. Susan is awake and fully aware of her surroundings. Her legally authorized representative is Tom, her husband. She has been approached by study staff to consider participation in the HABP/VABP trial that uses the early enrollment strategy with advance consent. The 2 study antibiotics, imipenem and meropenem, were referred to as Antibiotic A and Antibiotic B, respectively.

Opt-Out Procedures

As part of the story line in the vignette, we explained to patients and caregivers that including an opt-out process was found acceptable among key stakeholders given the context of the early enrollment strategy (in addition to participants' universal rights to opt out of any study at any time), and feedback was needed on how an opt-out approach might work. Participants were then asked to share their perspectives on 2 opt-out approaches within the vignette's story line:

Option 1: The study coordinator should confirm with Susan (the vignette patient) at the time she develops pneumonia that it is acceptable for study staff to proceed with giving her Antibiotic A or Antibiotic B.

© 2018 Corneli A et al. *JAMA Network Open.*

Option 2: Study staff should automatically proceed with giving Susan Antibiotic A or Antibiotic B, given her prior consent, unless she voices that she no longer wants to take part in the study.

IRB representatives were given the same options, although the questions were not framed within a vignette.

Precedent Autonomy

In the interviews with patients and caregivers, we continued with the vignette-based approach to frame questions, although we introduced a change to the scenario. In the vignette, Susan provides advance consent to participate in the HABP/VABP trial. Soon after providing consent, she becomes unconscious and is placed on a machine that helps her breathe. She remains unconscious for a week, and at the end of that week she develops pneumonia. We asked participants to identify the best option for proceeding with randomization: In Option A, her legally authorized representative, Tom, would decide whether Susan should be randomized. In Option B, randomization would proceed because Susan previously provided consent. In Option C, Susan would receive standard treatment because she is unable to opt out of randomization. Option D would be another approach (to be suggested by the participant).

The same vignette described above was also used in the interviews with IRB representatives. In the vignette, Susan, a patient in the ICU, provided advance consent to participate in the HABP/VABP trial. Soon after providing consent, she becomes incapacitated and is placed on a ventilator. She remains incapacitated for a week, and at the end of that week she develops VABP. IRB representatives were then given the same options as described above for patients and caregivers, to explore their opinions on the best approach.

© 2018 Corneli A et al. *JAMA Network Open.*
